# IL-1β-Mediated Activation of Adipose-Derived Mesenchymal Stromal Cells Results in PMN Reallocation and Enhanced Phagocytosis: A Possible Mechanism for the Reduction of Osteoarthritis Pathology

**DOI:** 10.3389/fimmu.2019.01075

**Published:** 2019-05-27

**Authors:** Stephanie C. M. van Dalen, Arjen B. Blom, Birgitte Walgreen, Annet W. Slöetjes, Monique M. A. Helsen, Edwin J. W. Geven, Menno ter Huurne, Thomas Vogl, Johannes Roth, Fons A. J. van de Loo, Marije I. Koenders, Louis Casteilla, Peter M. van der Kraan, Martijn H. J. van den Bosch, Peter L. E. M. van Lent

**Affiliations:** ^1^Experimental Rheumatology, Department of Rheumatology, Radboud University Medical Center, Nijmegen, Netherlands; ^2^Institute of Immunology, University of Münster, Münster, Germany; ^3^STROMALab, University of Toulouse, Toulouse, France

**Keywords:** CiOA, synovitis, adipose-derived mesenchymal stromal cells, interleukin-1β, chemokines, PMNs, phagocytosis

## Abstract

**Background:** Injection of adipose-derived mesenchymal stromal cells (ASCs) into murine knee joints after induction of inflammatory collagenase-induced osteoarthritis (CiOA) reduces development of joint pathology. This protection is only achieved when ASCs are applied in early CiOA, which is characterized by synovitis and high S100A8/A9 and IL-1β levels, suggesting that inflammation is a prerequisite for the protective effect of ASCs. Our objective was to gain more insight into the interplay between synovitis and ASC-mediated amelioration of CiOA pathology.

**Methods:** CiOA was induced by intra-articular collagenase injection. Knee joint sections were stained with hematoxylin/eosin and immunolocalization of polymorphonuclear cells (PMNs) and ASCs was performed using antibodies for NIMP-R14 and CD271, respectively. Chemokine expression induced by IL-1β or S100A8/A9 was assessed with qPCR and Luminex. ASC-PMN co-cultures were analyzed microscopically and with Luminex for inflammatory mediators. Migration of PMNs through transwell membranes toward conditioned medium of non-stimulated ASCs (ASC_NS_-CM) or IL-1β-stimulated ASCs (ASC_IL-1β_-CM) was examined using flow cytometry. Phagocytic capacity of PMNs was measured with labeled zymosan particles.

**Results:** Intra-articular saline injection on day 7 of CiOA increased synovitis after 6 h, characterized by PMNs scattered throughout the joint cavity and the synovium. ASC injection resulted in comparable numbers of PMNs which clustered around ASCs in close interaction with the synovial lining. IL-1β-stimulation of ASCs *in vitro* strongly increased expression of PMN-attracting chemokines CXCL5, CXCL7, and KC, whereas S100A8/A9-stimulation did not. In agreement, the number of clustered PMNs per ASC was significantly increased after 6 h of co-culturing with IL-1β-stimulated ASCs. Also migration of PMNs toward ASC_IL-1β_-CM was significantly enhanced (287%) when compared to ASC_NS_-CM. Interestingly, association of PMNs with ASCs significantly diminished KC protein release by ASCs (69% lower after 24 h), accompanied by reduced release of S100A8/A9 protein by the PMNs. Moreover, phagocytic capacity of PMNs was strongly enhanced after priming with ASC_IL-1β_-CM.

**Conclusions:** Local application of ASCs in inflamed CiOA knee joints results in clustering of attracted PMNs with ASCs in the synovium, which is likely mediated by IL-1β-induced up-regulation of chemokine release by ASCs. This results in enhanced phagocytic capacity of PMNs, enabling the clearance of debris to attenuate synovitis.

## Background

Osteoarthritis (OA) affects various tissues within the joint and is characterized by destruction of cartilage, formation of osteophytes and eventually disability. Additionally, in joints of many OA patients synovial inflammation is evident (1, 2), which could be ignited by several factors including damage-associated molecular patterns (DAMPs) (3), crystals (4), or cartilage components (5). Synovitis is likely important for removal of this tissue debris, thereby promoting repair processes (6), but on the other hand, synovial inflammation may also aggravate joint destruction via prolonged release of pro-inflammatory factors and cartilage-degrading enzymes (7).

Bone marrow (BM)-derived mesenchymal stromal cells (MSCs) exhibit immunosuppressive effects, and are therefore seen as a potential therapeutic tool for various inflammatory diseases (8, 9). Because adipose-derived mesenchymal stromal cells (ASCs) share multiple properties with BM-derived MSCs, are more abundant (5% of nucleated cells in adipose tissue vs. 0.0001–0.001% in BM), and can more easily be obtained, adipose tissue is an attractive alternative source of multipotent MSCs (10).

Over the recent years, several clinical trials have been conducted to treat OA patients with ASCs (11). In multiple proof-of-concept trials, including several dose-escalation studies, no severe adverse events or complications were found (12–15). The first phase I dose-escalating clinical trial, ADIPOA, showed that ASC injection is safe and well-tolerated in patients with knee osteoarthritis, and provided encouraging preliminary evidence of efficacy (16). The mechanism by which ASCs reduce joint pathology remains largely unknown. Recently we described that local application of ASCs in murine knee joints with early collagenase-induced OA (CiOA) ameliorated end stage joint destruction (17). Moreover, ASCs rapidly suppressed synovial thickening in early CiOA, and already 6 h after intra-articular injection, synovial release of key pro-inflammatory factors S100A8/A9 and interleukin (IL-)1β had significantly diminished. Cytokine levels remained low throughout the course of the disease measured up to day 42 after induction of the CiOA model (18).

This suppressive effect was only found when ASCs were applied early after induction of CiOA when synovitis was substantial, but not when administered at a later phase when synovitis was much lower (17). Cartilage protection after early ASC injection was confirmed in the mild medial meniscal destabilization model of OA in rabbits (19). Correspondingly with these studies, intra-articular application of ASCs early after induction of the destabilization of the medial meniscus (DMM) model, a surgical model of OA in which synovial inflammation is scant, had no effect on development of joint destruction (18). These findings indicate that the protective effect of ASCs is promoted by synovitis.

Many studies have suggested that ASCs directly suppress inflammation and tissue pathology solely by producing anti-inflammatory factors (20). However, previous results from our lab suggest that other mechanisms may also be relevant. We found that ASCs associate with the inflamed synovium within 24 h after intra-articular injection, but no more ASCs were detected 5 days after injection (17). Furthermore, a recent phase I clinical trial reported that a low dose of intra-articularly injected ASCs reduced pain levels and improved joint function (16). Based on these findings we hypothesized that early application of ASCs in inflammatory OA joints results in a rapid, potent protection, which remains effective up till later stages.

The aim of the present study was to investigate the underlying mechanism of ASC-mediated amelioration of joint destruction during CiOA. We started off with a histological analysis of CiOA joints shortly after intra-articular injection of ASCs. These *in vivo* observations provided the basis for further examination *in vitro*. We analyzed the result of ASC stimulation with inflammatory factors which are present in early CiOA on their chemokine production, as well as their physical interactions with immune cells. Additionally, to identify a possible mechanism by which ASCs may interrupt the vicious cycle of inflammation and joint destruction in CiOA, the immunomodulatory effects of ASCs on the attracted immune cells were determined.

## Methods

### Animals

Female C57BL/6NRj mice were obtained from Janvier. Mice were used at the age of 12 weeks old and were housed in filter-top cages with corncob bedding under standard conditions. A standard diet and tap water was available *ad libitum*. Group sizes were based on expected variation and a power of 80%. All animal studies were according to the Dutch law and were approved by the local Animal Experimentation Committee (RU-DEC 2013-215).

### Induction of CiOA

CiOA was induced by two unilateral, intra-articular injections of 1 U collagenase type VII from *Clostridium histolyticum* (Sigma-Aldrich) into knee joints of 16 C57BL/6NRj mice on days 0 and 2, causing damage to collateral and cruciate ligaments leading to local instability of the knee joint. This resulted in an OA-like phenotype with chronic synovial activation and cartilage destruction as was presented before in the article that initially described this OA-model (21). Day 42 was taken to model end point of the disease. Contralateral saline-injected knee joints were used as controls.

### ASC Isolation and Culture

Murine ASCs were isolated from the heterogeneous crude stromal fraction of adipose tissue surrounding the inguinal lymph nodes of C57BL/6NRj donor mice, by digestion of the fat tissue with Collagenase A (Roche) and selection by adhesion onto plastic overnight. ASCs were cultured according to standard procedures in DMEM/F12 (Gibco) supplemented with 10% newborn calf serum (Sigma-Aldrich), 1% penicillin/streptomycin (Invitrogen), 0.5% amphotericin B (Invitrogen), 16 μM biotin (Sigma-Aldrich), 18 μM pantothenic acid (Sigma-Aldrich), and 100 μM ascorbic acid (Sigma-Aldrich). Purity of the population was checked by flow cytometry analysis of presence (>80%) of cell markers Sca-1, CD44, CD105, and absence (<5%) of CD11b, c-Kit, and CD34, as described earlier (17).

Human ASCs were isolated from the stromal vascular fraction from liposuctions as previously described (16). Briefly, after collagenase digestion of the fat tissue (NB6, Coger), the stromal vascular fraction was seeded and selected by adhesion onto plastic overnight. Cells were cultured according to standard procedures in MEM (MacoPharma, Tourcoing) supplemented with human platelet growth factor-enriched plasma, 10 μg/mL ciprofloxacin, and 1 U/mL heparin. Purity of the population was checked by flow cytometry analysis for presence of cell markers CD73 (>90%), CD90 (>90%), and CD105 (>80%) and absence of cell markers CD14 (<2%), CD34 (<10%), and CD45 (<2%). All cells used in experiments were <passage 3.

### Intra-articular ASC Injection

On day 7 after induction of CiOA, 20,000 ASCs in 6 μL saline supplemented with 1% bovine serum albumin (BSA) fraction V (Sigma-Aldrich), were injected intra-articularly in both knees of 8 mice. Eight mice with CiOA were injected bilaterally with only saline supplemented with 1% BSA as control. In a separate experiment, three groups of 22 mice with CiOA were injected intra-articularly with either 20,000 ASCs or 100,000 ASCs in 6 μL saline supplemented with 1% BSA fraction V, or only saline supplemented with 1% BSA as control, on day 7 after induction of the model. Before injection, the viability of the ASCs was determined using trypan blue (Sigma-Aldrich) uptake and no cell death was observed.

### Immunohistochemistry

Both left and right total knee joints of 16 mice were collected 6 h after ASC injection, as well as the right joints with CiOA of 33 mice on days 14 and 42 after induction of the model. Joints were fixed in 4% formalin, decalcified in 4% formic acid buffered in phosphate-buffered saline (PBS) and embedded in paraffin. Coronal sections (7 μm) were stained with hematoxylin/eosin (HE). Immunostaining of total knee joints was performed to visualize the presence of polymorphonuclear cells (PMNs) with the specific antibody NIMP-R14 (kind gift from M. Strath, National Institute for Medical Research, London, UK). Briefly, after deparaffinization and antigen retrieval with trypsin for 5 min, sections were stained for 1 h with NIMP-R14 antibody, followed by horseradish peroxidase-conjugated rabbit anti-rat antibody for 1 h. PMN influx was quantified separately as infiltrate in the synovium and exudate in the joint cavity, which was then combined to an average total PMN influx (arbitrary score, 0–3). The localization of ASCs was visualized with antibodies against CD271 (AP07713PU-N, OriGene). Briefly, after deparaffinization and antigen retrieval in citrate buffer, sections were stained for 1 h with CD271 antibody, followed by biotinylated anti-rabbit antibody for 30 min and horseradish peroxidase-conjugated avidin–biotin complexes for 30 min. All sections were developed with diaminobenzidine (Sigma-Aldrich) and counterstained with hematoxylin.

### *In vitro* ASC Stimulation

Murine and human ASCs were plated in a 24 wells plate (80% confluency) and after adhesion overnight, cells were stimulated for 24 h with 1 μg/ml S100A8 or S100A9 [produced in our facilities (22)], various concentrations of IL-1β (0.1, 1, and 10 ng/ml) [murine: kind gift from I. G. Otterness (Pfizer Central Research, Groton, CT); human: R&D Systems], or a combination. 100 ng/ml LPS was used as a control for the presence of Toll-like receptor (TLR)4 on the cell membrane of ASCs.

### Preparation of RNA and Quantitative Real-Time Polymerase Chain Reaction (qRT-PCR)

Stimulated cells were lysed in TRI-reagent (Sigma-Aldrich) followed by RNA isolation according to the manufacturer's protocol. The RNA was reverse transcribed into cDNA as previously described (23). mRNA levels of chemokines were detected using a StepOnePlus qRT-PCR system (Applied Biosystems) using SYBRgreen master mix (Applied Biosystems) and specific primers (Biolegio; Table 1). Relative quantification of the qRT-PCR signals was performed by correcting the C_t_ value of the gene of interest for glyceraldehyde 3-phosphate dehydrogenase (GAPDH) expression (–ΔC_t_).

**Table 1:** Primers used for qRT-PCR.

**Gene**	**Forward primer**	**Reverse primer**
mGAPDH	5′-GGCAAATTCAACGGCACA-3′	5′-GTTAGTGGGGTCTCGCTCCTG-3′
mCXCL5	5′-GCTCCTGTGATAAAGAAAATCATTCA-3′	5′-CGAGTGCATTCCGCTTAGCT-3′
mCXCL7	5′-CACTGTGCTGATGTGGAAGTGATAG-3′	5′-TTTGGGTCCAGGCACGTTT-3′
mKC	5′-TGGCTGGGATTCACCTCAA-3′	5′-GAGTGTGGCTATGACTTCGGTTT-3′
hGAPDH	5′-ATCTTCTTTTGCGTCGCCAG-3′	5′-TTCCCCATGGTGTCTGAGC-3′
hIL-8	5′-AGAAGTTTTTGAAGAGGGCTGAGA-3′	5′-CAGACCCACACAATACATGAAGTG-3′

### Measurement of Protein Levels in Culture Supernatants

Culture supernatants were collected to analyze protein levels. The release of keratinocyte chemoattractant (KC) and IL-8 were measured using Luminex multianalyte technology, using the Bio-Rad Bio-Plex™ 200 System with specific magnetic beads. Protein levels of S100A8/A9 in these supernatants was quantified by a sandwich enzyme-linked immunosorbent assay (ELISA) specific for murine S100A8/A9 as described previously (24).

### Clustering Assay

Murine primary bone marrow (BM)-PMNs were freshly isolated from femurs of C57BL/6NRj mice with MACS microbeads specific for Ly6B.2 (Miltenyi Biotec) according to the manufacturer's protocol. Murine ASCs were plated in a 24 wells plate (50% confluency), and after adhesion overnight, freshly isolated BM-PMNs were added in a 1:10 ratio and cultured with or without 1 ng/ml IL-1β in 50% ASC medium and 50% PMN medium [RPMI (Gibco) supplemented with 10% fetal calf serum (Sigma-Aldrich) and 1% penicillin/streptomycin (Invitrogen)]. After 3, 6, 24, and 48 h supernatants were collected for measurement of protein release upon clustering. Moreover, after 6 h, the culture medium was removed and non-adherent cells were vigorously washed away during the several steps of fixing and staining the attached cells in the plate with May-Grünwald Giemsa (MGG). The number of clustering cells was quantified in pictures taken at five separate areas in the well using ImageJ 1.46r software. A cluster was defined as an adherent ASC with at least one PMN attached on its surface.

### Preparation of Conditioned Medium

Conditioned medium (CM) from ASCs was collected after 24 h culture without stimulation (ASC_NS_-CM), or after IL-1β stimulation (ASC_IL-1β_-CM). In the latter case ASCs were stimulated for 24 h with 1 ng/ml IL-1β, washed twice with saline to remove all exogenous IL-1β, and cultured for another 24 h with fresh ASC culture medium before collection. CM was filtered before use in the migration and phagocytosis assays described below and stored at −20°C when not used immediately.

### Migration Assay

ASC_NS_-CM was supplemented with 100 ng/ml KC (R&D Systems) as positive control, and with 25 ng/ml monocyte chemotactic protein 1 (MCP-1) or 1 ng/ml IL-1β as negative controls. CMs were added to the lower compartment of 3 μm transwell inserts (Costar). 500,000 freshly isolated primary BM-PMNs in ASC_NS_-CM were placed in the top compartment of the transwell inserts and incubated for 2 h. Migrated cells in the lower compartment were collected and quantified with flow cytometry after addition of 123count eBeads™ Counting Beads (Thermo Fisher Scientific).

### Phagocytosis Assay

Freshly MACS-isolated primary murine BM-PMNs and human blood PMNs (MACSxpress® Whole Blood Neutrophil Isolation Kit, Miltenyi Biotec) were incubated at 37°C for 1 h with ASC_NS_-CM, ASC_IL-1β_-CM (without exogenous IL-1β), or fresh ASC culture medium as negative control. After washing with PBS, cells were incubated with pHrodo™ Red Zymosan BioParticles® (Life Technologies) at 37°C for 1 h. Next, the phagocytic capacity was assessed by measuring fluorescence intensity (FI) using a Clariostar Monochromator Microplate Reader (BMG LABTECH). The FI was corrected for the average background fluorescence measured in triplicate in samples containing no cells.

### Statistical Analysis

Statistical analyses were performed using Graphpad Prism version 5.03. Differences between groups were tested using a Student's *t*-test or a one-way analysis of variance (ANOVA), followed by a Dunnett's posttest or a Bonferroni Multiple Comparison posttest. *P*-values lower than 0.05 were considered significant. Results are expressed as mean values ± standard deviation (SD).

## Results

### PMNs Reallocate and Cluster With ASCs in Knee Joints With Early CiOA After Intra-articular ASC Injection

**Figure 1:**
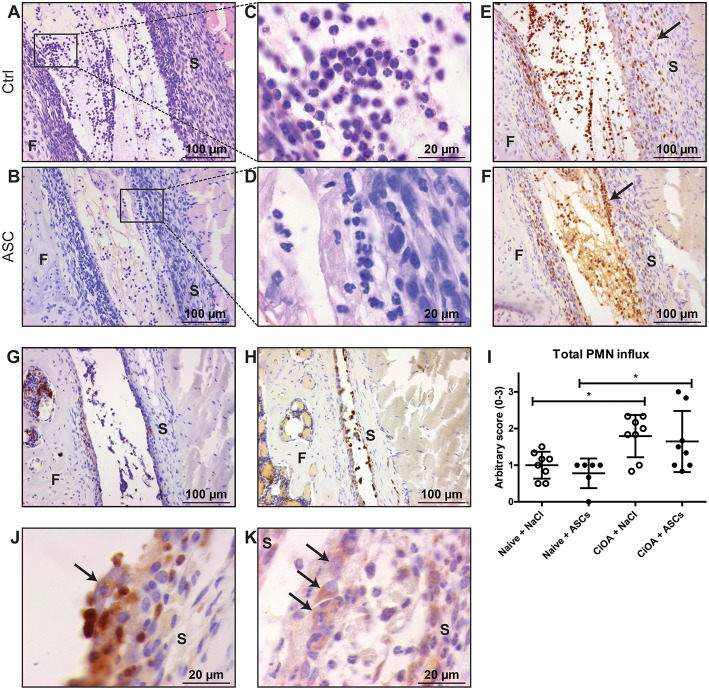
PMNs reallocate and cluster with ASCs in knees with early CiOA after intra-articular ASC injection. **(A,B)** Intra-articular injection on day 7 of CiOA resulted in attraction of immune cells within 6 h, both in the control **(A)** and ASC-injected **(B)** joints, as was shown in HE-stained total knee joint sections. **(C,D)** Higher magnifications showed that the immune cells in both saline- **(C)** and ASC-injected **(D)** CiOA knee joints had a polymorphonuclear (PMN) phenotype. **(E,F)** Immunohistochemistry with the specific antibody NIMP-R14 confirmed that a large number of PMNs is attracted to the joints. They were equally spread throughout the synovium and the joint cavity in the control-injected joints **(E)**, but in the ASC-injected joints a remarkable accumulation of PMNs along the lining was found **(F)** as indicated by arrows. **(G)** NIMP-R14 staining showed that 24 h after ASC injection the PMN influx had largely disappeared. **(H)** ASC injection in naïve joints resulted in NIMP-R14 positive cell influx, albeit lower than in CiOA joints. **(I)** Quantification of attracted PMNs after intra-articular injection confirmed a significantly increased number of PMNs in CiOA joints compared to naïve joints. **(J)** At higher magnification we observed a clustering of PMNs around cells we supposed to be ASCs (arrow), which was confirmed with a CD271 antibody **(K)** which specifically stains ASCs (arrows). Control joints were CiOA knees injected with saline supplemented with 1% BSA. Images shown are representative for the treatment groups (*n* = 8 per group). Original magnification × 200 **(A,B,E,F,G,H)** or × 1,000 **(C,D,J,K)**. Differences between groups were tested using a one-way ANOVA followed by a Bonferroni Multiple Comparison posttest. F, femur; S, synovium. Bars show mean values ± SD. *P < 0.05.

To investigate the acute ameliorating effects of ASCs, we injected 20,000 ASCs in murine knee joints on day 7 after induction of CiOA, when synovitis is high. An increased number of immune cells was observed within the joint cavity 6 h after intra-articular ASC injection (Figure 1B). Interestingly, this cell influx was also found in the saline-injected control CiOA knee joints (Figure 1A). Morphological screening of HE-stained sections showed that the majority of the attracted cells in both saline- and ASC-injected CiOA knee joints had a PMN phenotype (Figures 1C,D) which was confirmed by immune staining using the PMN-specific antibody NIMP-R14. We observed that injection of saline caused an influx of PMNs scattered throughout the joint (Figure 1E). Interestingly, after injection of ASCs the attracted PMNs were found in aggregates along the synovial lining layer (Figure 1F). Twenty four hours after ASC injection, the number of PMNs was greatly reduced in both the saline- and ASC-injected CiOA joints, suggesting that the reallocation of PMNs is short-lasting (Figure 1G). ASC injection in naïve joints resulted in less PMN influx and no accumulation of cells nearby the synovium (Figure 1H). Quantification of the attraction of PMNs showed that both ASC and control injections result in a significantly elevated influx of PMNs in CiOA knees compared to naïve joints (Figure 1I). This indicates that an injection in an already inflamed OA knee joint in itself causes a rapid flare leading to a transient attraction of immune cells. Interestingly, at higher magnification we observed a clustering of PMNs around cells we supposed to be ASCs based on their size (arrow in Figure 1J), which was confirmed with immunolocalization of ASCs using an antibody against the specific marker CD271 (Figure 1K). These *in vivo* results possibly designate an underlying mechanism for ASC-mediated joint protection. Therefore they were a prelude for the following *in vitro* studies in which we investigated the clustering of PMNs with ASCs in more detail to gain insight into this possible mechanism.

### Expression of PMN-Attracting Chemokines by ASCs Is Elevated by IL-1β *in vitro*

**Figure 2:**
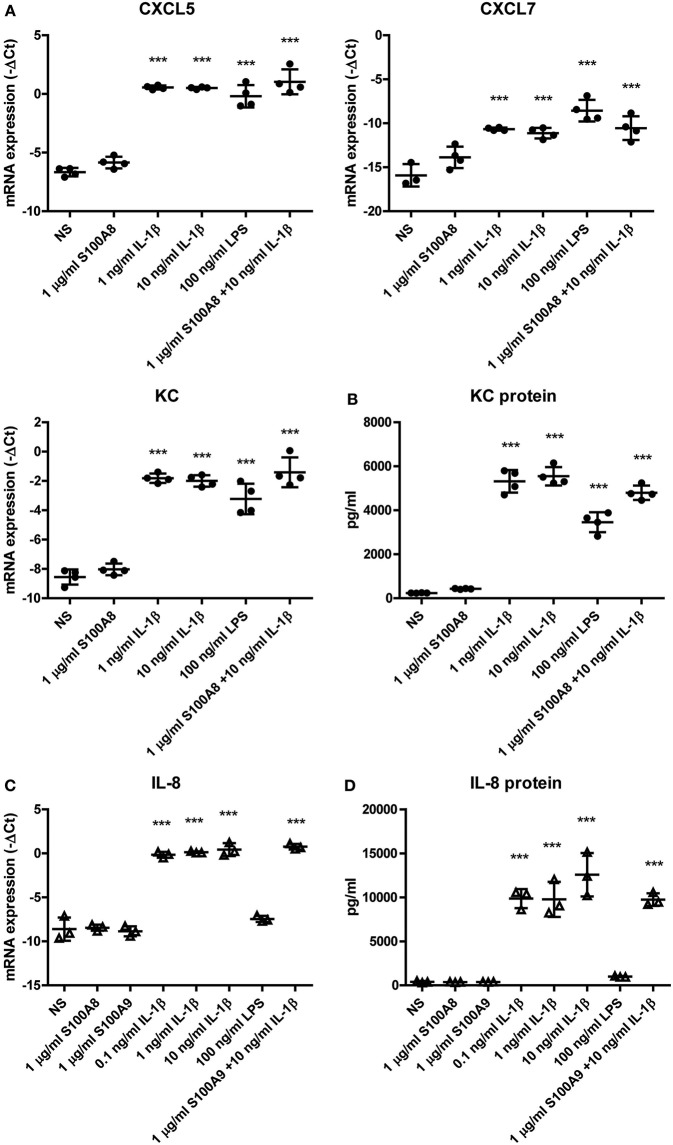
Expression of PMN-attracting chemokines by ASCs is elevated by IL-1β *in vitro*. The effect of an inflammatory milieu on gene expression in ASCs was assessed by 24 h stimulation with different pro-inflammatory mediators. **(A)** Murine ASCs showed a significantly increased gene expression of PMN-attracting chemokines CXCL5, CXCL7, and KC after stimulation with IL-1β. **(B)** Also on protein level in the supernatant, KC was significantly up-regulated after IL-1β stimulation. Human ASCs show a comparable elevated level of the functional KC-homolog IL-8 after IL-1β stimulation on both gene expression **(C)** and protein level **(D)**. Gene expression levels are presented as –ΔC_*t*_ compared to GAPDH. Closed circles represent murine samples, open triangles represent human samples [*n* = 4 **(A,B)** or 3 **(C,D)** per group]. Differences between non-stimulated (NS) ASCs and the several stimulations were tested using a one-way ANOVA followed by a Dunnett's posttest. Bars show mean values ± SD. ***P < 0.001 vs. NS ASCs.

First, we investigated whether the apparent preference of PMNs to co-localize with the ASCs is induced by inflammatory mediators that are present during inflammatory OA. Therefore, we determined if S100A8 and IL-1β, which are predominantly elevated during the early stage of CiOA, enhanced the production of the PMN-attracting chemokines C-X-C motif chemokine ligand 5 (CXCL5), CXCL7, and KC by murine ASCs. A significant elevation in gene expression of these chemokines was found after 24 h stimulation with IL-1β (Figure 2A), with a maximal increase after stimulation with only 1 ng/ml IL-1β (70, 38, and 107, fold increase for CXCL5, CXCL7, and KC, respectively, *P* < 0.001). KC showed the strongest up-regulation of gene expression and is generally accepted as the main PMN-attracting chemokine, which typically shows a very specific and potent attraction of PMNs. Therefore, we determined protein levels of KC as a representative PMN-attracting chemokine in culture supernatants and found a 22-fold increase after stimulation with only 1 ng/ml IL-1β (Figure 2B). Unexpectedly, stimulation with high levels of S100A8 (1 μg/ml) did not enhance chemokine gene expression or protein levels. Moreover, a combination of S100A8 and IL-1β did not alter the effect of IL-1β.

To extrapolate our findings in murine ASCs to the human situation, we next stimulated ASCs obtained from human adipose tissue for 24 h with S100A8, S100A9, IL-1β, or a combination of S100A9 and IL-1β, to confirm that ASC-mediated attraction of PMNs is also plausible to be present in ASC-injected OA patients. Like in murine ASCs, low levels of IL-1β (0.1 ng/ml) already gave a significant 355-fold increase in gene expression (Figure 2C) and a 26-fold increase in protein levels (Figure 2D) of IL-8, the human functional homolog of KC (*P* < 0.001). In contrast, high levels of S100A8 and S100A9 (1 μg/ml) did not elevate IL-8 levels.

### Clustering of PMNs With ASCs Is Enhanced by IL-1β *in vitro*

**Figure 3:**
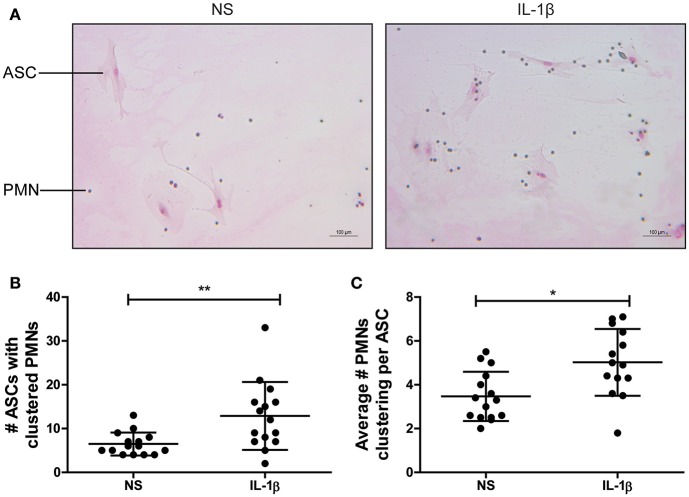
Clustering of murine PMNs with ASCs is enhanced by IL-1β *in vitro*. The association of freshly isolated murine PMNs with adherent ASCs driven by IL-1β was assessed in a co-culture experiment. **(A)** After 6 h, clustering cells were stained with May-Grünwald Giemsa and quantified. Large cells are ASCs, small cells are PMNs. Both the number of ASCs that formed clusters with PMNs **(B)** and the average number of PMNs clustered per ASC **(C)** were significantly increased in presence of IL-1β when compared to the non-stimulated co-cultures (NS). *N* = 3 per group, quantified at five separate areas per sample. Differences between the groups were tested using a Student's *t*-test. Bars show mean values ± SD. *P < 0.05, **P < 0.01.

To further study the tendency of the attracted PMNs to cluster with ASCs, murine primary PMNs and adherent murine ASCs were co-cultured without stimulation (NS) or with 1 ng/ml IL-1β. After 6 h, PMNs (small cells in Figure 3A) were clearly clustered with ASCs (large cells in Figure 3A). Analysis of the number of ASCs with clustered PMNs showed a significant increase of 99% after co-culture in the presence of IL-1β (*P* < 0.01) (Figure 3B). Moreover, the average number of PMNs clustering per individual ASC was significantly increased with 45% after 6 h of co-culture with IL-1β (*P* < 0.05). These data demonstrate that IL-1β promotes an interaction between ASCs and PMNs (Figure 3C).

### PMN Attraction by ASCs Is Increased by IL-1β *in vitro*

**Figure 4:**
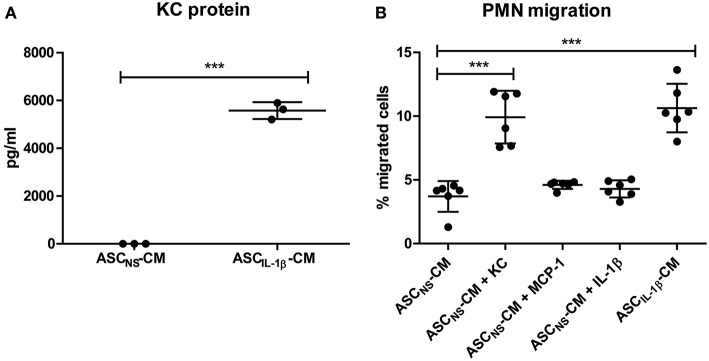
PMN attraction by ASCs is increased by IL-1β *in vitro*. The potential of IL-1β-stimulated ASCs to attract PMNs was investigated using transwell inserts. **(A)** KC levels in conditioned medium (CM) of IL-1β-stimulated ASCs (ASC_IL-1β_-CM, no exogenous IL-1β present) were significantly elevated compared to CM of non-stimulated ASCs (ASC_NS_-CM). **(B)** Migration of freshly isolated PMNs toward ASC_IL-1β_-CM as well as toward the positive control KC was significantly increased compared to ASC_NS_-CM. No enhanced migration of PMNs was found in the negative controls containing MCP-1 or IL-1β. The number of migrated PMNs was expressed as percentage of total cells added to the top compartment [*n* = 3 **(A)** or 6 **(B)** per group]. Differences between groups were tested using a Student's *t*-test **(A)** or a one-way ANOVA followed by a Dunnett's posttest **(B)**. Bars show mean values ± SD. ***P < 0.001 vs. ASC_NS_-CM.

*In vivo* the PMNs need to be actively attracted to the ASCs, before they are able to cluster. Because IL-1β strongly enhanced KC expression by ASCs, we investigated the attraction of PMNs *in vitro* with conditioned medium of non-stimulated ASCs (ASC_NS_-CM) and ASCs that were stimulated with IL-1β for 24 h (ASC_IL-1β_-CM). Luminex measurements confirmed that protein levels of KC in ASC_IL-1β_-CM were strongly increased when compared to ASC_NS_-CM (5,576 vs. 5.33 pg/ml) (Figure 4A). Using transwell inserts we found that CM of IL-1β-stimulated ASCs significantly increased the migration of PMNs by 287% (from 3.7 to 10.6%), confirming that ASCs attract PMNs upon IL-1β-stimulation (Figure 4B). Likewise, ASC_NS_-CM supplemented with KC as positive control showed an increase of PMN migration by 268% (from 3.7 to 9.9%). In contrast, the migration of PMNs toward ASC_NS_-CM was low (<5%), comparable to the negative controls MCP-1 and IL-1β alone.

### IL-1β-Mediated Release of Inflammatory Factors Is Significantly Lowered After Clustering of PMNs With ASCs

**Figure 5:**
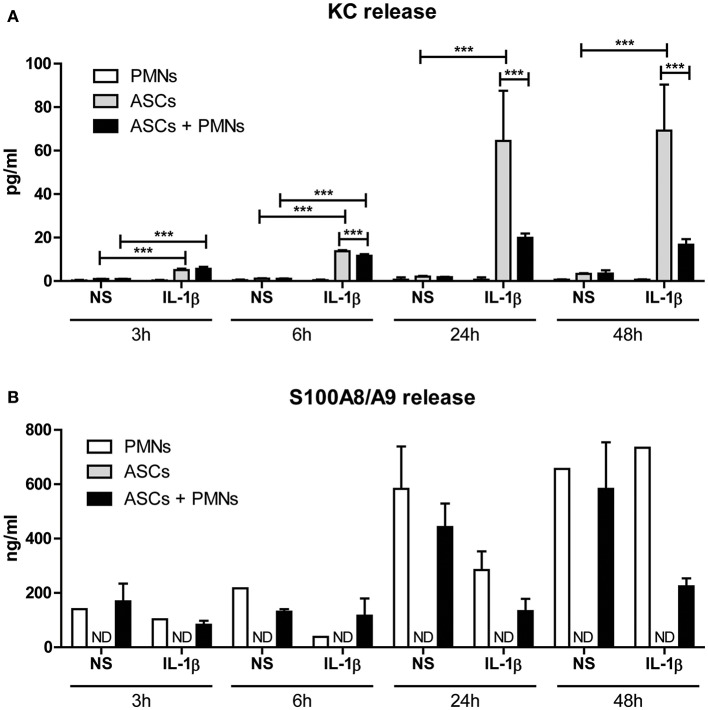
IL-1β-mediated release of inflammatory factors is significantly lowered after clustering of PMNs with ASCs. The effect of clustering of PMNs with ASCs on protein release was determined. **(A)** KC release by ASCs was already significantly increased after 3 and 6 h of co-culture with PMNs in presence of IL-1β. After 24 and 48 h of co-culture with IL-1β, clustering of PMNs with ASCs resulted in a significantly lowered KC release when compared to ASCs cultured alone. **(B)** S100A8/A9 release was not detectable in a monoculture of ASCs, but PMNs released basal levels of S100A8/A9. These levels increased over time, but remained lower in co-cultures of PMNs with ASCs in presence of IL-1β. *N* = 3 per group. Differences between groups were tested using a one-way ANOVA followed by a Bonferroni Multiple Comparison posttest for each time point separately. Bars show mean values ± SD. ***P < 0.001. ND, not detectable.

As attraction of PMNs and clustering with ASCs in presence of IL-1β was evident, we next determined the effect on the inflammatory activity of these cells by analyzing the release of KC protein after co-culture. Only minimal amounts of KC were released by PMNs cultured alone in the presence of IL-1β, indicating that ASCs are the main source of KC production. When no IL-1β was added, no KC was released in all co-cultures (Figure 5A), underlining that IL-1β is a potent inducer of PMN-attracting chemokine release by ASCs. In the presence of IL-1β, protein levels of KC in the supernatant of ASCs were already significantly elevated after 3 h (*P* < 0.001). KC levels strongly increased to maximal elevation (up to 65 ng/ml) after 24 h of stimulation with IL-1β which remained at the same level after 48 h. Interestingly, 24 h of co-culturing PMNs with ASCs in presence of IL-1β resulted in significantly lower KC release by ASCs (69 and 76% lower after 24 and 48 h incubation, respectively) (Figure 5A), suggesting that IL-1β-driven chemokine production by ASC is rapidly inhibited after clustering of PMNs. Furthermore, S100A8/A9 release by PMNs was not altered in the presence of IL-1β or in co-culture with ASCs after 3 and 6 h (Figure 5B). However, after 24 and 48 h we found strongly elevated S100A8/A9 release. S100A8/A9 release by a monoculture of ASCs with or without IL-1β stimulation was undetectable. Interestingly, in the presence of IL-1β-stimulated ASCs PMNs released lower levels of S100A8/A9 when compared to non-stimulated ASCs.

### Phagocytosis by PMNs Is Enhanced After Priming With Conditioned Medium of IL-1β-Stimulated ASCs

**Figure 6:**
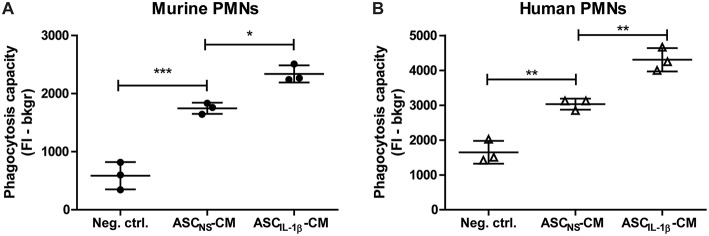
Phagocytosis by PMNs is enhanced after priming with conditioned medium of IL-1β-stimulated ASCs. The immunomodulatory effect of ASCs on PMNs was investigated by assessment of the phagocytic capacity after priming with ASC-conditioned medium. **(A)** The uptake of labeled zymosan particles by murine PMNs primed with conditioned medium (CM) of non-stimulated ASCs (ASC_NS_-CM) was significantly enhanced when compared to the negative control containing fresh culture medium. Incubation of PMNs with CM of ASCs which were stimulated with IL-1β (ASC_IL-1β_-CM, no exogenous IL-1β is present) further enhanced phagocytosis when compared to ASC_NS_-CM. **(B)** Human PMNs primed with ASC_NS_-CM showed a similar increase in phagocytic capacity when compared to culture medium, which was even further enhanced after priming with ASC_IL-1β_-CM. Closed circles represent murine samples, open triangles represent human samples (*n* = 3 per group). Differences between groups were tested using a one-way ANOVA followed by a Bonferroni Multiple Comparison posttest. Bars show mean values ± SD. *P < 0.05, **P < 0.01, ***P < 0.001.

To gain insight in the possible way in which the attraction and clustering of PMNs contributes to the amelioration of OA pathology, we finally investigated whether IL-1β-activated ASCs could enhance removal of tissue debris by inducing phagocytosis. Already after 1 h of priming murine PMNs with ASC_NS_-CM, ingestion of fluorescent zymosan particles was significantly increased to 198% (*P* < 0.001) when compared to the negative control containing only culture medium. Interestingly, priming PMNs with ASC_IL-1β_-CM resulted in an even further enhanced phagocytic capacity of 34% (*P* < 0.05) compared to ASC_NS_-CM (Figure 6A). We next set out to demonstrate that these results can be translated from the murine to the human situation by using human PMNs which were primed with CM of human ASCs. Again, we found a significant up-regulation of 84% (*P* < 0.01) in the phagocytic capacity of PMNs primed with ASC_NS_-CM, which was further increased with an additional 42% (*P* < 0.01) after priming with ASC_IL-1β_-CM (Figure 6B). Our findings show that IL-1β-stimulated ASCs not only attract PMNs, but also increase their phagocytic activity thereby possibly promoting tissue repair.

## Discussion

In the present study we find that local application of ASCs in inflamed CiOA knee joints leads to rapid clustering of PMNs around ASCs, which may designate a mechanism by which ASCs reduce OA pathology. *In vitro* we uncover that IL-1β stimulates this clustering which reduces the pro-inflammatory activity of the PMNs. At the same time the phagocytic capacity of PMNs is enhanced, which may substantially resolve inflammation and potentially accelerates joint repair processes.

ASCs are known for their regenerative capacities that enable the repair of damaged tissues such as cartilage (25), but they also possess immunosuppressive characteristics (20, 26–28). Previously, we found that ASCs rapidly suppressed synovitis and development of joint destruction when locally administered to inflamed OA joints (17, 18). However, the exact working mechanism of ASCs remained unknown, albeit several noteworthy observations have been published. Firstly, a previous study demonstrated that human ASCs applied in knee joints of normal SCID mice may reside at the site of injection up to 6 months (29). Contrarily, we showed that murine ASCs injected into an inflamed CiOA joint first home to the synovium within 24 h but disappear quickly thereafter (17). The presence of inflammation may explain the enhanced ASC clearance in CiOA knee joints. Pro-inflammatory mediators like IL-1β promote the migratory behavior of ASCs by elevating chemokine release and expression of adhesion molecules such as CD49d (integrin α4), and CD54 (ICAM-1) (30–32). Secondly, injection of numbers as low as only 20,000 ASCs more effectively protected against synovial inflammation in CiOA joints than a 5 times higher dose as shown in Supplementary Figure 1. This finding was confirmed in a recent phase I clinical trial that reported reduced pain levels and improved joint function after an intra-articular injection of a low dose of ASCs (16). Finally, ASCs only suppressed joint damage when applied in an inflammatory environment (17, 18). These three crucial observations make it unlikely that ASCs exclusively protect directly against joint damage in our murine OA model via production of anti-inflammatory mediators. This led to our hypothesis that early application of ASCs in inflammatory OA joints results in a rapid, potent protection, which remains effective up till later stages. This protection can be achieved by interactions of ASCs with the immune system, causing a powerful and long-lasting amplification of their immunosuppressive capacity.

Our hypothesis is supported by the strikingly different localization of PMNs 6 h after intra-articular injection. The saline injection flared the smoldering synovial inflammation, resulting in recruitment of PMNs that scattered throughout the joint, whereas application of ASCs induced association of PMNs with ASCs into aggregates that resided particularly on top of the inflamed synovial lining layer. That this interaction indeed has the potency to shift the inflammatory activity of ASCs and PMNs was reflected by the reduced KC and S100A8/A9 release, respectively, in our *in vitro* co-culture experiments. Previous studies showed that synovial lining macrophages are crucial in mediating S100A8/A9-driven synovitis during experimental OA (33, 34). The physical interaction of immunomodulatory ASC-PMN aggregates with the synovial lining may explain why synovial S100A8/A9 protein levels were already significantly reduced between 6 and 48 h after intra-articular ASC injection on day 7 of CiOA (18).

Next to the altered release of inflammatory mediators, ASCs can simultaneously direct PMNs to effectively ameliorate ongoing inflammation in OA joints via enhanced phagocytic activity. Apart from the clearance of debris, phagocytosis also causes PMNs to become apoptotic. This leads to ingestion by macrophages, which in turn start to release anti-inflammatory and reparative cytokines thereby inducing tissue repair (35). CM from non-stimulated ASCs already augmented phagocytosis by PMNs, which was further increased by IL-1β-stimulation of ASCs. The potential effect of ASCs on PMNs was underlined in a murine sepsis model, where ASC-induced phagocytosis by PMNs resulted in enhanced bacterial clearance and lowered mortality (36). In a different study, microarray analysis demonstrated that MSC treatment of mice with the cecal ligation and puncture model of sepsis resulted in enriched pathways for Fc receptor–mediated phagocytosis, natural killer cell signaling, and antigen presentation (37).

Phagocytosis is likely to be a crucial process in our CiOA model as collagenase injections damage collateral ligaments leading to mechanical instability of the joint (38). Insufficient removal of tissue debris prolongs synovitis thereby contributing to cartilage degradation and ectopic bone formation (21). Enhanced removal of tissue debris by ASC-stimulated PMNs may reduce the inflammatory response and simultaneously clear the area for more effective repair of ligaments. This would explain why we previously found less joint destruction in end stage CiOA after ASC injection (17). PMN attraction alone is not sufficient to reduce joint damage, since control injections without ASCs induced similar PMN influx but did not protect the joint from developing cartilage and bone destruction later on (17, 18). This supports our idea that ASCs applied in an inflammatory environment not only reallocate but also activate PMNs to protect the joint from further destruction.

In search of soluble factors that stimulate ASCs to attract PMNs, we focused on two pro-inflammatory mediators that characterize synovitis in early CiOA. Firstly, the contribution of the important TLR4 ligand S100A8/A9 to joint destruction during CiOA is well-described (34, 39). Elevated levels of S100A8/A9 have also been measured in OA patients (34, 40). A previous study showed that after stimulation with the TLR4 ligand LPS MSCs produce inflammatory mediators such as IL-1β, IL-6, and IL-8, indicating the expression of TLR4 on the cell membrane of ASCs (41, 42). Unexpectedly, S100A8/A9 did not induce the expression of KC or IL-8 by ASCs *in vitro*. This difference could be explained by the observation that TLR4 signaling by S100A8/A9 is different from that of LPS since S100A8/A9 signaling is independent of CD14 (24).

Secondly, we focused on IL-1β that is elevated in synovium and cartilage in OA patients (2, 43). Although the role of IL-1β in human OA is still a matter of debate (44), we recently showed that IL-1β does not contribute to CiOA pathology (45, 46). IL-1β directly induces the immunosuppressive phenotype of ASCs via activation of NF-κB and JNK signaling pathways (47) thereby stimulating several paracrine effects (48, 49). In the current study we found that very low levels of IL-1β strongly enhance gene and protein expression of PMN-attracting chemokines KC in murine cells and IL-8 in human cells, enabling a powerful amplification of PMN reallocation and clustering with ASCs. This in turn enables the ASC-mediated increase in phagocytosis *in vitro*, and possibly also *in vivo*. These results emphasize that although IL-1β does not aggravate joint destruction in CiOA, it may be a crucial factor for the ASC-mediated protection against joint destruction that was published before (16–18, 50). It also explains why ASC treatment is only effective when applied early in inflammatory CiOA (17), as elevated IL-1β levels are only present during early stages (34). ASC treatment after induction of CiOA in IL-1β^−/−^ mice would be an elegant experiment to confirm the crucial role of IL-1β.

The ultimate goal is to treat human OA patients with ASCs. In line with previous mouse studies (17, 18, 27), a phase 1 human trial showed that a single injection of a low number of ASCs into knee joints of OA patients ameliorated pain and MRI analysis of ASC-injected joints indicates reduced development of joint destruction (16). Currently, a double blind phase IIb study called ADIPOA2 is performed in which 150 OA patients will be treated intra-articularly with autologous ASCs.

## Conclusions

In our study we demonstrate that IL-1β-stimulated ASCs reallocate inflammatory PMNs thereby inducing cluster formation with ASCs, resulting in increased phagocytic capacity. We found this not only with murine, but also with human cells. These results may explain the previously published reduction in OA pathology after ASC injection into an inflamed joint. This more extensive knowledge of how intra-articular injection of ASCs initiates the protective effect, and particularly which factors drive this process, enables the prediction of the efficacy of ASC treatment. This paves the way for the development of well-defined inclusion criteria for OA patients.

## Ethics Statement

All animal studies were according to the Dutch law and were approved by the local Animal Experimentation Committee (RU-DEC 2013-215).

## Author Contributions

SvD, AB, FvdL, MK, PvdK, MvdB, and PvL: conception and design of study. SvD, BW, AS, MH, EG, MtH, TV, and JR: acquisition of data. SvD, AB, MtH, MvdB, and PvL: analysis and interpretation of data. TV, JR, and LC: contribution of reagents, materials, analysis tools. SvD, AB, MvdB, and PvL: drafting the article. SvD, AB, BW, AS, MH, EG, MtH, TV, JR, FvdL, MK, LC, PvdK, MvdB, and PvL: final approval of the submitted manuscript.

### Conflict of Interest Statement

The authors declare that the research was conducted in the absence of any commercial or financial relationships that could be construed as a potential conflict of interest.

## Supplementary Material

Supplementary Figure 1 20,000 ASCs protect more efficiently against synovial inflammation in CiOA than 100,000 ASCs. On day 7 after induction of CiOA, two groups of 22 mice were injected intra-articularly with either 20,000 ASCs or 100,000 ASCs in 6 μL saline supplemented with 1% bovine serum albumin (BSA) fraction V. Twenty-two mice with CiOA were injected with only saline supplemented with 1% BSA as control. On day 14 and 42, total knee joints with CiOA were collected and stained with hematoxylin/eosin (HE) to score synovial inflammation which was identified as thickening of the synovium (arbitrary score from 0 to 3, three sections per knee joint). Intra-articular injection of 20,000 ASCs resulted in significantly lower synovial inflammation in CiOA than injection of 100,000 ASCs on both day 14 and day 42. *N* = 11 per group. Differences between groups were tested using a one-way ANOVA followed by a Bonferroni Multiple Comparison posttest. Bars show mean values ± SD. ^*^*P* < 0.05.

## Abbreviations

ANOVA: one-way analysis of variance
ASC: adipose-derived mesenchymal stromal cell
BM: bone marrow
BSA: bovine serum albumin
CD: cluster of differentiation
cDNA: complementary DNA
CiOA: collagenase-induced OA
CM: conditioned medium
C_t_: cycle threshold
CXCL: C-X-C motif chemokine ligand
DAMP: damage-associated molecular pattern
DMM: destabilization of the medial meniscus
ELISA: enzyme-linked immunosorbent assay
FI: fluorescence intensity
GAPDH: glyceraldehyde 3-phosphate dehydrogenase
HE: hematoxylin/eosin
ICAM-1: intercellular adhesion molecule-1
IL: interleukin
JNK: c-Jun N-terminal kinase
KC: keratinocyte chemoattractant
LPS: lipopolysaccharide
MCP-1: monocyte chemotactic protein 1
MGG: May-Grünwald Giemsa
mRNA: messenger RNA
MSC: mesenchymal stromal cell
NF-κB: nuclear factor κB
ND: not detectable
NS: no stimulation
OA: osteoarthritis
PBS: phosphate-buffered saline
PMN: polymorphonuclear cell
qRT-PCR: quantitative real-time polymerase chain reaction
SD: standard deviation
TLR: Toll-like receptor
U: unit.
