# Supplementary figures and images for: IL-1β-Mediated Activation of Adipose-Derived Mesenchymal Stromal Cells Results in PMN Reallocation and Enhanced Phagocytosis: A Possible Mechanism for the Reduction of Osteoarthritis Pathology
Source: Front Immunol. 2019 May 27;10:1075. doi: 10.3389/fimmu.2019.01075 (PMC6545928; doi:10.3389/fimmu.2019.01075)

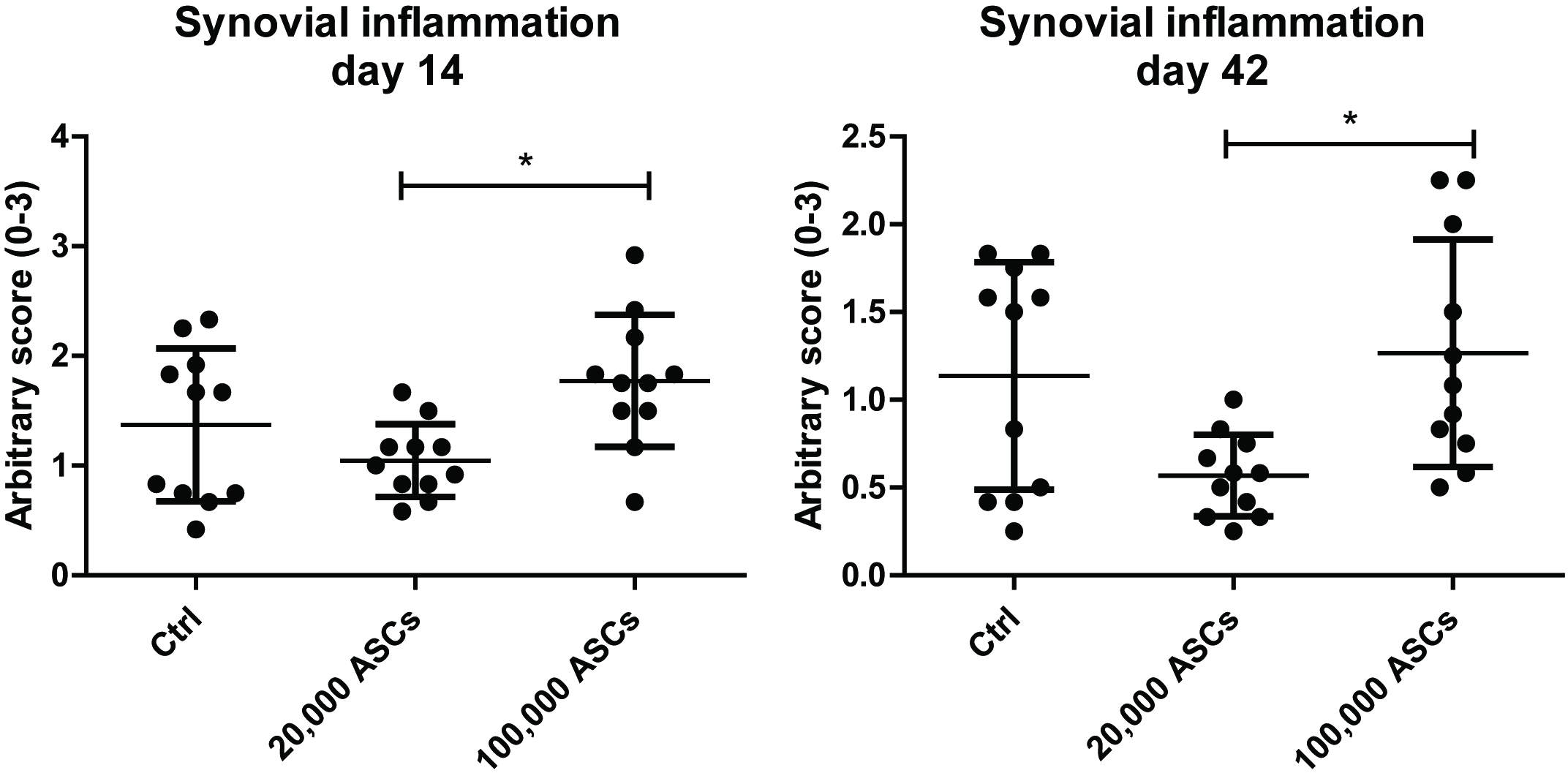

Supplement: Supplementary Figure 1 — 20,000 ASCs protect more efficiently against synovial inflammation in CiOA than 100,000 ASCs. On day 7 after induction of CiOA, two groups of 22 mice were injected intra-articularly with either 20,000 ASCs or 100,000 ASCs in 6 μL saline supplemented with 1% bovine serum albumin (BSA) fraction V. Twenty-two mice with CiOA were injected with only saline supplemented with 1% BSA as control. On day 14 and 42, total knee joints with CiOA were collected and stained with hematoxylin/eosin (HE) to score synovial inflammation which was identified as thickening of the synovium (arbitrary score from 0 to 3, three sections per knee joint). Intra-articular injection of 20,000 ASCs resulted in significantly lower synovial inflammation in CiOA than injection of 100,000 ASCs on both day 14 and day 42. N = 11 per group. Differences between groups were tested using a one-way ANOVA followed by a Bonferroni Multiple Comparison posttest. Bars show mean values ± SD. *P < 0.05. [file Image_1.TIF]
